# Arousal system stimulation and anesthetic state alter visuoparietal connectivity

**DOI:** 10.3389/fnsys.2023.1157488

**Published:** 2023-04-17

**Authors:** Axel Hutt, Anthony G. Hudetz

**Affiliations:** ^1^MLMS, MIMESIS, Université de Strasbourg, CNRS, lnria, ICube, Strasbourg, France; ^2^Department of Anesthesiology, Center for Consciousness Science, University of Michigan, Ann Arbor, MI, United States

**Keywords:** general anesthesia, local field potentials, synchronization, brainstem, stimulation

## Abstract

Cortical information processing is under the precise control of the ascending arousal system (AAS). Anesthesia suppresses cortical arousal that can be mitigated by exogenous stimulation of the AAS. The question remains to what extent cortical information processing is regained by AAS stimulation. We investigate the effect of electrical stimulation of the nucleus Pontis Oralis (PnO), a distinct source of ascending AAS projections, on cortical functional connectivity (FC) and information storage at mild, moderate, and deep anesthesia. Local field potentials (LFPs) recorded previously in the secondary visual cortex (V2) and the adjacent parietal association cortex (PtA) in chronically instrumented unrestrained rats. We hypothesized that PnO stimulation would induce electrocortical arousal accompanied by enhanced FC and active information storage (AIS) implying improved information processing. In fact, stimulation reduced FC in slow oscillations (0.3–2.5 Hz) at low anesthetic level and increased FC at high anesthetic level. These effects were augmented following stimulation suggesting stimulus-induced plasticity. The observed opposite stimulation-anesthetic impact was less clear in the γ-band activity (30–70 Hz). In addition, FC in slow oscillations was more sensitive to stimulation and anesthetic level than FC in γ-band activity which exhibited a rather constant spatial FC structure that was symmetric between specific, topographically related sites in V2 and PtA. Invariant networks were defined as a set of strongly connected electrode channels, which were invariant to experimental conditions. In invariant networks, stimulation decreased AIS and increasing anesthetic level increased AIS. Conversely, in non-invariant (complement) networks, stimulation did not affect AIS at low anesthetic level but increased it at high anesthetic level. The results suggest that arousal stimulation alters cortical FC and information storage as a function of anesthetic level with a prolonged effect beyond the duration of stimulation. The findings help better understand how the arousal system may influence information processing in cortical networks at different levels of anesthesia.

## 1. Introduction

Cortical information processing is under the precise control of the ascending arousal system (AAS) in both wakefulness and sleep (Parvizi and Damasio, 2001; Lee and Dan, 2012). Recent investigations reveal that the AAS not only maintains cortical arousal but also participates in shaping cognitive functions. For example, visuospatial attention tasks are optimized by activity of the AAS (Wainstein et al., 2021) and influence the awareness of cognitive states (Munn et al., 2021).

Cortical projections of the AAS originate in the brainstem with relays in the midline thalamus, basal forebrain, hypothalamus (Jones and Yang, 1985; Holstege and Kuypers, 1987). A distinct source of ascending projections is the oral part of the pontine reticular nucleus (PnO) (Moruzzi and Magoun, 1949; Jones and Yang, 1985; Jones, 2003). Anesthesia suppresses AAS activity and arousal along these pathways (Mashour et al., 2005; Brown et al., 2011; Lee and Dan, 2012) including those originating from the PnO (Sukhotinsky et al., 2006).

Conversely, pharmacological stimulation of various sites of the AAS can mitigate the suppression of arousal in both sleep and anesthesia. For example, microinjection of the acetylcholinesterase inhibitor neostigmine or the cholinergic agonist carbachol into the pontine reticular formation enhances rapid-eye-movement-sleep-like state in rodent models (Bourgin et al., 1995; Lydic et al., 2002). Stimulation of the mesencephalic reticular formation in lightly anesthetized animals produces electrocortical activation and visual stimulus-induced synchronization (Munk et al., 1996). Electrocortical and behavioral suppression by various anesthetics can be mitigated by stimulation of the intralaminar thalamus (Alkire et al., 2007), basal forebrain (Pillay et al., 2011; Dean et al., 2022), ventral tegmental area (Solt et al., 2014), parabrachial nucleus (Muindi et al., 2016), and the PnO (Pillay et al., 2014a).

While AAS stimulation can restore signs of wakefulness in anesthetized animals, the question remains to what extent information processing is regained by AAS stimulation. Investigating the effect of AAS stimulation on neuronal communication as indexed by neuronal functional connectivity (FC) may help provide the answer. We previously investigated the effect of brainstem stimulation on neuronal interaction complexity estimated from unit recordings in rats (Pillay et al., 2014b). Here we extend this work to local field potentials (LFPs) that represent the activity of neuronal populations involved in cortical information processing. We utilize previously recorded LFP data from the secondary visual area (V2) and the adjacent parietal association cortex (PtA) in rats at three levels of anesthesia. The cortical areas selected for recording are thought to be involved in information integration and they were previously seen to be modulated by electrical stimulation of the PnO (Pillay et al., 2014b). Several levels of anesthesia were examined given its potentially nonlinear effect on neuronal population activity. We compared the LFP spectral content and FC of 16 recording sites before, during and after brainstem stimulation at each anesthetic level. We hypothesized that stimulation would induce electrocortical arousal and increase FC in the gamma frequency band implying improved information exchange among the recording sites. As we will show, our hypothesis was partially supported, although in a qualitatively unexpected way.

## 2. Materials and methods

### 2.1. Physiological experiment and protocol

**FIGURE 1:**
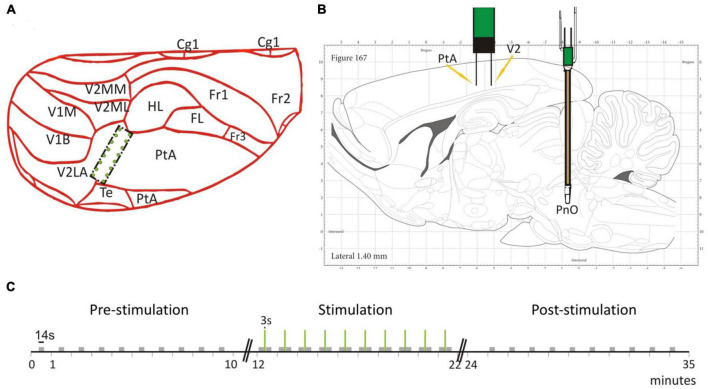
Electrode placement and stimulation protocol. **(A)** The 2 × 8 electrode grid was implanted on the border of the parietal association cortex (PtA) and the secondary visual cortex (V2). The schematic is overlaid on a partial dorsal view of the rat brain. **(B)** The brainstem stimulation was applied in the pontine reticular nucleus (PnO). Schematic is overlaid on a sagittal view of the rat brain. **(C)** Temporal stimulation protocol with PnO stimulation marked as green vertical lines. Modified from Figure 1 in Pillay et al. (2014b) with permission.

**FIGURE 2:**
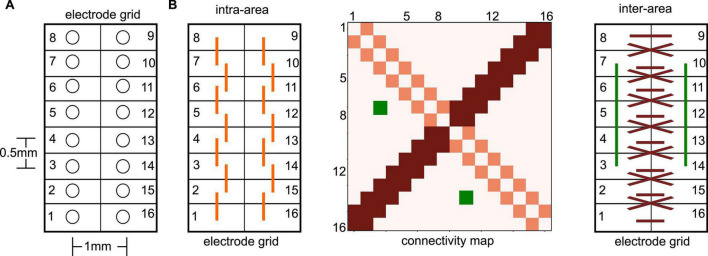
Illustration of electrode grid and related connectivity map. **(A)** The electrode numbers increase on one side from the bottom to the top and further increase on the other grid side from top to bottom in a circle. This configuration implies opposite neighboring sites of, e.g., #1 and #16. It is important to mention that electrode grids have been positioned in the neural tissue in such a way, that each grid side captured activity from a single brain area. **(B)** Extracted connectivity maps (like the illustrated map in the center panel) exhibit certain patterns due to the spatial electrode locations in the electrode grid. For instance, nearest neighbor connections on the same grid side (left panel), i.e., the same brain area, are seen in the connectivity map (center panel) on the diagonal (light red). Symmetric nearest neighbor connections between opposite electrodes sites (right panel) show up in the connectivity map on the second diagonal (dark red). Symmetric non-diagonal connections (e.g., the green-coded connectivity map pixels) reflect connections symmetric on both grid sides.

We present experimental data from seven adult male, Sprague-Dawley rats. To record LFPs, an electrode array consisting of 16 microwires was implanted at the border of the PtA and the secondary visual cortex (V2), cf. Figure 1A. The microwire array had two rows in a 2 × 8 configuration with row spacing of 0.5 mm and row separation of 1 mm (Figure 2A). The brainstem area PnO was stimulated by a concentric bipolar electrode (Figure 1B). For more details, see Pillay et al. (2014b).

For testing, the rats were placed into a custom-built, transparent experimental box where they retained the ability to breathe spontaneously throughout the experiment. Desflurane and O_2_ (30%) were delivered at a flow rate of 5 L/min and rat body temperature was controlled at 37°C throughout the duration of the experiment. We mention that desflurane is known to cause concentration-dependent suppression of spontaneous respiration (Kapoor and Vakamudi, 2012). We carefully monitored the animals’ chest movement under anesthesia to see if their breathing patterns were impaired. No adverse respiratory effect happened in our experiments and motion-related artifacts were not observed in the anesthetized rats. Moreover, we did not see any related gamma amplitude modulation at the respiratory rate. PnO stimulation parameters were guided by previous work (Antognini et al., 2003) and a stimulation block consisted of a 3 s train of 0.1 ms pulses delivered at 300 Hz at a current intensity of 0.8 mA (Figure 1C). The stimulus strength was insufficient to restore consciousness as assessed by behavioral signs. Before stimulation, the righting reflex, a surrogate index of consciousness in rodents (Franks, 2008), was weakly present at low desflurane concentration but was absent at medium and high concentrations. Stimulation at medium or high anesthetic concentrations produced LFP desynchronization but no gross motor behavior including any attempt for righting. The stimulation session lasted 10 min and 10 stimulation blocks were applied with constant inter-stimulus intervals of 57 s. Each stimulation session was proceeded by a pre-stimulation and post-stimulation session, when no stimulation was applied. In addition, each rat was tested under three desflurane concentrations in random order: 3.5% (in the following called *low anesthetic level*), 4.5% (*medium anesthetic level*), and 6% (*high anesthetic level*). These concentrations were chosen since the rat’s righting reflex, a surrogate measure for loss of consciousness (Franks, 2008), is believed to occur within this range. As tested in our experiments, the righting reflex was barely present at the low anesthetic level and was absent at the medium anesthetic level. At the high anesthetic level, rats were under deep anesthesia. The order of anesthetic concentrations was randomized from rat to rat. We point out, that the anesthetic concentration was kept constant during the pre-stimulation, stimulation and post-stimulation phase. Randomization of anesthetic concentrations was applied to minimize both positive and negative carryover effects. Because of the relatively long, 20 min equilibration time between different anesthetic concentrations, hysteresis was unlikely. In fact, in our previous study comparing the effect of increasing and decreasing desflurane concentrations, no evidence of hysteresis was found (Hudetz et al., 2020).

### 2.2. Data processing

Local field potentials and time markers from electrical stimuli were recorded with sampling rate 1 kHz and bandpass filtered between 0.3 and 500 Hz. Afterward, each LFP channel (electrode time series) has been notch-filtered offline at the power line frequency 60 Hz and down-sampled to 500 Hz. This leads to *N* = 3 ⋅ 10^5^ sampling points in the pre-stimulation and post-stimulation time series. Since we have been interested in the LFPs stationary activity, in each electrode channel we removed the artifacts evoked by PnO stimulation by cutting out data from 400 ms before stimulation onset and 2 s after stimulation offset.

This reduced the number of sampling points to *N* = 297,300 in the stimulation condition. In addition, stimulation-independent artifacts have occurred, and they were removed by replacing artifact values by the time series temporal average when the LFP deviation from temporal average exceeded five times the time series standard deviation.

### 2.3. Spectral and synchronization measures

The power spectral density (PSD) of the data was computed by the Bartlett-Welch method with a sliding window of 10 s and the window overlap rate of 0.9. In case of band-limited prominent PSD of time series, it is reasonable (Rosenblum et al., 2000) to compute and interpret phase values in these frequency bands. The degree of synchronization between two time series *s*_1_(*t*_*n*_), *s*_2_(*t*_*n*_), *n* = 1*,…,N* may be expressed as the *resultant length R* (Mardia, 1972) of the time series’ instantaneous phase differences △ *ϕ_*n*_* = ϕ_1_(*t*_*n*_) − ϕ_2_(*t*_*n*_). Assuming a time interval [*t*_*m*_;*t*_*m*_ + *T*] of *T* data points, the corresponding results length is defined as


$$R_{m}=\frac{1}{T}\,\sqrt{{\left(\sum_{n=m}^{m+T-1}\cos\Delta \phi_{n}\right)}^{2}+{\left(\sum_{n=m}^{m+T-1}\sin\Delta \phi_{n}\right)}^{2}.}$$

The instantaneous phases ϕ_1_(*t*_*n*_),ϕ_2_(*t*_*n*_) of time series may be computed by wavelet transform (Lachaux et al., 1999) or Hilbert transform (Le Van Quyen et al., 2001) in a certain frequency band. Then the corresponding synchronization index is defined as the *Phase Locking Value* (Lachaux et al., 2002) *PLV* = *R*_1_ with *T* = *N* and 0 ≤ PLV ≤ 1: if *PLV* = 0 then two time series are not synchronized or not coherent, whereas *PLV* = 1 reflects full synchronization or maximum coherence.

In this context, it is important to point out that instantaneous phases are defined properly and can be interpreted in a certain frequency band only if the signals power spectrum exhibits a narrow-band peak in this frequency band (Rosenblum et al., 2000). In the presented data, we have identified spectral peaks about 1 and 50 Hz only and consequently our analysis will focus on slow oscillations in the frequency interval [0.3;2.5 Hz] and on γ-activity in the interval [30;70 Hz]. The PLV quantifies the degree of phase synchronization between two time series, where it considers the difference of time series’ phase values. The Phase Locking Value (PLV) is a kind of inverse variance of the phase distribution about a mean phase: the smaller the variance of the phase distribution is the more similar are the phases in the distribution and the higher is the PLV and thus the stronger is the phase synchronization. The phase synchronization indices PLV are defined for two time series of two LFP channels and were computed in the respective frequency bands by a fourth-order Butterworth band pass filter followed by a Hilbert Transform of the two time series. This provided the instantaneous phase values *ϕ_*x*_*(*t*_*n*_) = arctan(Im(*A*_*n*_)/Re(*A*_*n*_)) by the corresponding complex-valued analytical signal *A*_*n*_ at time instance *t*_*n*_ in two time series (*x* = 1 and *x* = 2) with the imaginary and real part Im(*A*_*n*_) and Re(*A*_*n*_), respectively. The phase angle considered to compute the PLV is ϕ(*t*_*n*_) = *ϕ_1_*(*t*_*n*_)—*ϕ_2_*(*t*_*n*_). We point out that two time series can be strongly synchronized, i.e., have a large value of PLV, despite a non-zero mean phase difference. In such a case the mean phase angle reflects phase-lagged synchronization implying a non-zero delay between the channels. Moreover, it is important to mention that PLV-values quantify the degree of synchronization between two LFP channels and statistical distinctions between synchronized and non-synchronized states can only made in the context of statistical inference tests.

To ensure that the synchronization between two LFP channels is significantly non-zero, we have performed a permutation test with 200 random temporal permutations. The resulting *p*-values *p <* 10^–8^ have indicated significantly non-zero synchronization in all pairs assuming a significance level α = 0.05.

To understand better the synchronization in the channel pairs, we have estimated the kernel density of PLV-values over all channel pairs (Python 3.9 method *sklearn.neighbors.KernelDensity* with Epanechnikov-kernel type and band width 0.1). This density allows to identify rare (with low density values) weakly synchronized channel pairs (with low PLV-values). For each experimental condition and in each animal subject, we removed all channel pairs whose density undercut 20% of the maximum density value and whose PLV-values were smaller than the PLV-value with maximum density.

The remaining channel pairs may exhibit clusters of PLV-values reflecting subtle underlying connection networks. To extract such clusters, we have run a hierarchical clustering (Python 3.9 method *sklearn.cluster.AgglomerativeClustering* assuming Euclidean distances and single linkage cluster comparisons). A first attempt to detect the optimal number of clusters by the well-known gap statistic (Tibshirani et al., 2001) failed (it leads to the large number of ∼ 30 clusters, details not shown). Nevertheless, to gain a better idea on the distribution of synchronization over the electrode grid, we have assumed three clusters, which already provided some insights into the distribution. Sorting the three clusters by their median values lead to the following order: cluster *C*1 included lowest synchronization values and the strongest synchronized channel pairs were collected in cluster *C*3. Since channel pairs in cluster *C*1 are chosen to include weakly synchronized channel pairs, the channel pairs in this cluster *C*1 were excluded from the subsequent statistical evaluation and cluster *C*2- and *C*3-channel pairs were included only. Additional numerical studies with number of clusters 2, 4, and 5 have retained the qualitative results shown if the electrode pairs in the lowest cluster were removed from the analysis.

We assume in accordance with previous studies that synchronization between two electrode time series reflects FC between the underlying neural locations. Essentially, a detailed analysis of the connectivity patterns, i.e., the pattern of synchronized electrode pairs, indicated connection pairs symmetric to the counter-diagonal of the connectivity matrix, cf. Figure 2B, dark-red pixels and dark-red interactions. To quantify the degree of symmetry in the connections, we counted the number *N*_*s*_ of connections opposite to each other (e.g., the green connections in Figure 2), which belong to the same PLV-cluster *C*2 or cluster *C*3. The ratio of this number *N*_*s*_ and the total number of connection pairs *N*_*all*_ represents the degree of the connection symmetry *S* = *N*_*s*_*/N*_*all.*_

The FC patterns extracted under different experimental conditions allowed the identification of networks that emerge in all experimental conditions in each animal subject. We contended that due to their generality these networks reflect invariant interactions and they were identified for the slow frequencies and the γ-frequency band separately. Moreover, networks nodes which are part of invariant connections in both frequency bands, were defined to belong to an *invariant network*. Such a network was defined for each animal subject separately. The remaining network nodes were defined to belong to a *varying network*.

### 2.4. Active information storage

Stimulation and general anesthetics are known to affect information processing in the brain. Previous studies have shown significant modifications of information content under anesthesia (Wollstadt et al., 2017; Novelli and Lizier, 2021) supporting the Information Integration Theory of Tononi (2004). Active information storage (AIS) is the mutual information *I(.,.)* between a signal’s future state and its past state.


$$A\,I\,S\,\left(Y_{t}\right)=I\,\left(Y_{t};Y_{t-1}^{k}\right)$$

where *Y_t_* is the signal at time *t* and $Y_{t-1}^{k}\,\left(Y_{t-\tau },Y_{t-2\,\tau },\ldots ,Y_{t-k\,\tau }\right)$ is the past state of the last k time steps before time *t* and τ is the time delay between successive time steps (Lizier et al., 2012; Wollstadt et al., 2017). *AIS*(*Y*_*t*_) quantifies the amount of predictable information that is in use at time *t* for the next state update. Low values of AIS reflect processes that generate little information whereas high values of AIS indicate processes that visit many equi-probable states in a predictable sequence (Wollstadt et al., 2017). We utilized the Python package *IDTxl* (Wollstadt et al., 2018) with maximum delay time steps of *k* = 10; the delay time was τ = 2 ms and surrogate data were generated with 200 permutations utilizing the Kraskov implementation to estimate the implicit conditional mutual information (see Wollstadt et al. (2018) for more details). To compare the AIS between two experimental conditions, AIS was computed in a subset of channel time series in 20 subsequent time windows of 1 s duration each to gain 20 values of AIS. A channel subset comprised channels either from invariant or varying networks. Each AIS-value was compared statistically to a surrogate data distribution of 200 AIS values each gained by time permutations. We found that all AIS-values were significantly different from zero (*p* = 0.002 with Bonferroni-corrected α = 0.05/*N* where *N* ≤ 16).

## 3. Results

### 3.1. Global connectivity

**FIGURE 3:**
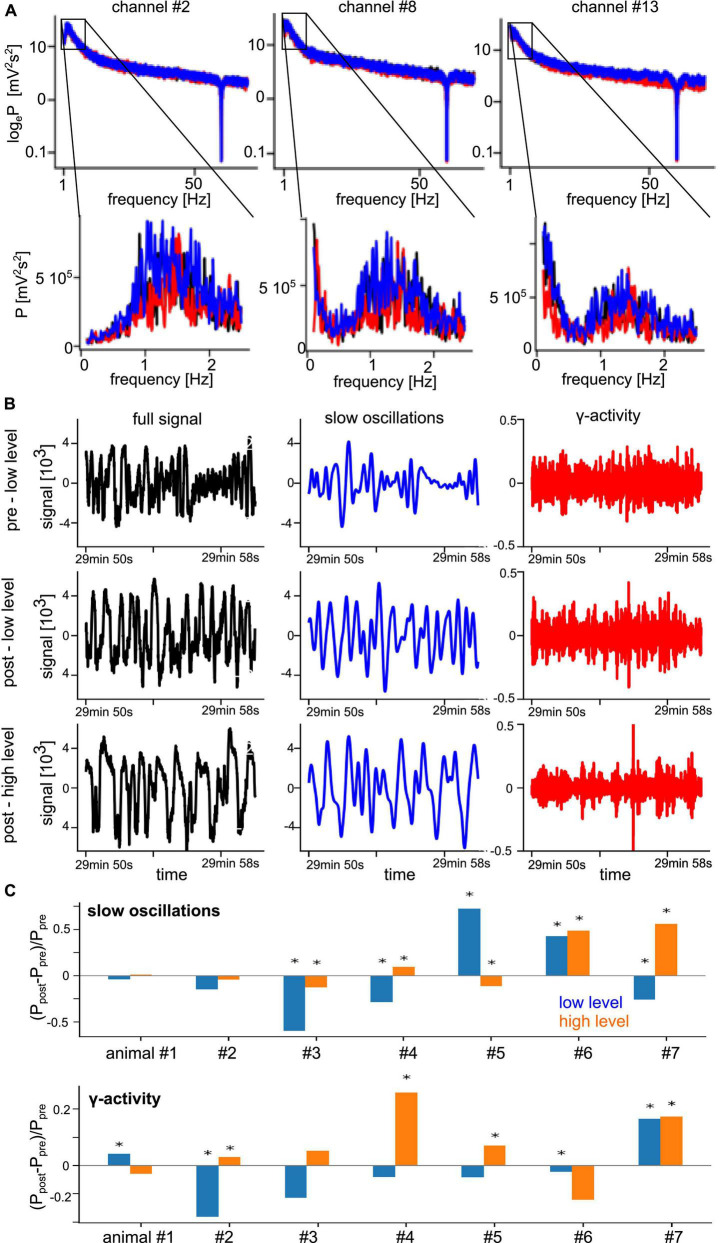
Prominent spectral activity at slow oscillations (∼1 Hz) and in the γ-frequency band. **(A)** Power spectral density of three selected electrode channels before stimulation (black line), during stimulation (red line), and after stimulation (blue line) from animal #7 at medium anesthesia level. **(B)** Time series of a typical signal (black), band pass-filtered signal about 1 Hz (blue), and γ-activity (red) in the pre- and post-stimulation phase at low anesthesia level (top row and center row) and in the post-stimulation phase at high anesthesia level (bottom row) from the same animal. **(C)** Relative change of global spectral power of slow oscillations (top panel) and γ-activity (bottom panel) before (P_*pre*_) and after (P_*post*_) stimulation at low (blue) and high (orange) anesthesia levels. Global power is defined as the median power value over all LFP channels in the respective frequency band. Significant changes between pre- and post-stimulation were tested by a pairwise non-parametric Wilcoxon test. *Significant differences are marked with asterisk (α = 0.05).

Functional connectivity in a brain network reflects the level of communication between network nodes. We consider the set of measurement electrodes as our network under study. The degree of phase synchronization between two measured time series was used to characterize the underlying neural networks’ FC (Bastos and Schoffelen, 2016). First, we identified the frequency bands which show non-negligible spectral power. Figure 3A shows typical power spectra of a few selected channels as an example from a single animal where we observe a clear spectral peak at slow oscillations with frequencies around 1.5 Hz. We also see that different stimulation conditions exhibit varying spectral power in the γ-frequency band at about 50 Hz. These observations are consistent across all experiments and conditions. Figure 3B presents LFP time series under different experimental conditions from the same electrodes. By visual inspection, we observe strong slow oscillation and low-magnitude γ-activity. In addition, the pre-stimulation and post-stimulation conditions under low anesthesia (top and center panels) exhibit similar magnitudes, whereas the high anesthesia level (bottom panel) shows a slightly enhanced signal. Investigating the change of global spectral power between pre- and post-stimulation (Figure 3C) sheds some light on the impact of stimulation and anesthesia. In the γ-activity, we observe a tendency of stimulation to diminish spectral power at low anesthesia level and enhance spectral power at high anesthesia level (Figure 3C, bottom panel). This insight follows from visual inspection but cannot be confirmed statistically due to the strong heterogeneity among animals. Spectral power of slow oscillations (Figure 3C, top panel) does not seem to reveal such a tendency.

**FIGURE 4:**
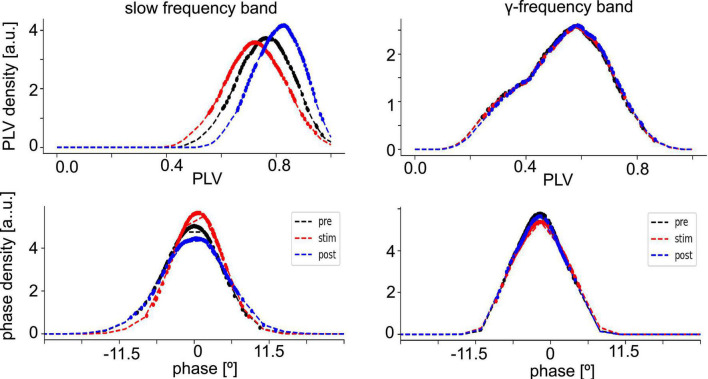
Distribution of phase synchronization. Phase Locking Value (PLV) density estimates **(top panels)** and phase difference density estimates **(bottom panel)** of all electrode pairs (dashed line). Dotted points denote data whose PLV density values is among the highest 80% of densities or have PLV-values larger than the PLVs with maximum density. The data are taken from animal #7 at medium anesthetic levels.

Since synchronization in the neural tissue may reflect the degree of interaction between neuronal populations, we have estimated the distribution of PLV values (phase synchronization index, see section “2. Materials and methods”) and corresponding phase differences at different stimulation conditions in both the slow oscillations and the γ-frequency band (Figure 4). The PLV-values of slow oscillations are slightly higher than those of the γ-frequency band, whereas the measured time series of both fluctuate around zero phase. This reflects a slightly stronger phase synchronization in slow oscillations than in γ-band activity.

After removal of electrode pairs with rare low PLV values (cf. section “2. Materials and methods”), in a first step, we attempted to identify important statistical factors in the set of PLV values by a two-way ANOVA for slow oscillations and the γ-frequency band (Table 1). The anesthetic level and the stimulation condition appear to have a certain impact on the synchronization of slow oscillations (*p <* 10^–8^), whereas they seem to exert negligible effect on γ-band synchronization (*p >* 0.05). Here, we assume a significance level of α = 0.05. Moreover, the synchronization depends on the animal subject (*p <* 10^–8^) and the anesthetic level and stimulation condition interact significantly (*p <* 0.01).

**TABLE 1:** Identification of significant factors.

Conditions	*F*-value	*p*-value	Conditions	*F*-value	*p*-value
**Level**	**33.78**	**<10^–8^**	Level	2.473	0.0844
**Stimulation**	**19.00**	**<10^–8^**	Stimulation	0.081	0.9200
**Animal**	**576.81**	**<10^–8^**	**Animal**	**199**	**< 10^–8^**
Level * stimulation	**8.31**	**1** ⋅ **10^–6^**	Level * stimulation	**3.33**	**0.0097**

Two-way ANOVA of Phase Locking Values (PLV) in channel pairs in connection cluster C2 and C3 for 3 factors and their interaction (⋅*⋅) for the slow oscillations (left) and the γ-frequency band (right). Experimental conditions and anesthetic level determine strongly synchronization in slow oscillations, whereas they have a poor impact in the γ-frequency band. The degrees of freedom are *df* = 2 (anesthetic level), *df* = 2 (stimulation condition), *df* = 6 (animals) and *df* = 4 (interaction of anesthetic level-stimulation). The significance level is α = 0.05 and statistically significant results are encoded in bold font.

To better understand how the synchronization depends on the anesthetic level and stimulation across the animals, we performed a two-way ANOVA test for each animal separately. Table 2 reveals that in most animals the anesthetic level and the stimulation condition have a significant impact on the slow oscillation synchronization. However, the interaction term level*stimulation is also significant (*p <* 10^–7^) in all animals indicating that the impact of anesthesia and stimulation depend on each other. This result suggests that there is an individual variability in the sensitivity of low-frequency oscillations to stimulation at specific levels. The impact of level and stimulation are less pronounced in the γ-frequency band (Table 2).

**TABLE 2:** Single animal factors in slow and γ-oscillations.

Animal	Condition	*F*-value	*p*-value	Animal	Condition	*F*-value	*p*-value
**Slow oscillations**							
#1	**Level**	**28**.**48**	<**10^–8^**	#5	**Level**	**251**.**69**	<**10^–8^**
	**Stimulation**	**7**.**96**	**0**.**0004**		Stimulation	2.59	0.075
	**Level × stimulation**	**9**.**74**	**1** ⋅ **10^–7^**		**Level × stimulation**	**34**.**20**	<**10^–8^**
#2	**Level**	**36**.**66**	<**10^–8^**	#6	**Level**	**25**.**27**	<**10^–8^**
	**Stimulation**	**12**.**83**	**3** ⋅ **10^–6^**		**Stimulation**	**29**.**90**	<**10^–8^**
	**Level × stimulation**	**8**.**71**	**7** ⋅ **10^–7^**		**Level × stimulation**	**17**.**88**	<**10^–8^**
#3	**Level**	**21**.**64**	<**10^–8^**	#7	**Level**	**61**.**50**	<**10^–8^**
	**Stimulation**	**20**.**31**	<**10^–8^**		**Stimulation**	**40**.**44**	<**10^–8^**
	**Level × stimulation**	**28**.**35**	<**10^–8^**		**Level × stimulation**	**50**.**81**	<**10^–8^**
#4	**Level**	**57**.**64**	<**10^–8^**				
	Stimulation	0.18	0.83				
	**Level × stimulation**	**17**.**51**	<**10^–8^**				
**γ-oscillations**							
#1	**Level**	**17.46**	<**10^–8^**	#5	Level	0.16	0.846
	**Stimulation**	**6.99**	**0**.**001**		Stimulation	0.20	0.814
	**Level × stimulation**	**9**.**2**	**3** ⋅ **10^–7^**		Level × stimulation	0.57	0.681
#2	**Level**	**3.45**	**0.031**	#6	**Level**	**10.61**	**0.044**
	Stimulation	2.57	0.077		**Stimulation**	**13.68**	**0.0219**
	Level × stimulation	1.92	0.105		Level × stimulation	5.78	0.0501
#3	**Level**	**5.92**	**0.002**	#7	level	**10.61**	**3.10^−5^**
	Stimulation	0.96	0.385		**Stimulation**	**13.68**	**10^−6^**
	Level × stimulation	7.37	**8.10^−6^**		Level × stimulation	5.78	**10^−4^**
#3	**Level**	**4.89**	0.008				
	**Stimulation**	**3.55**	**0.029**				
	Level × stimulation	1.24	0.29				

Two-way ANOVA of Phase Locking Values (PLV) in channel pairs in connection cluster C2 and C3 with degrees of freedom *df* = 2 (anesthetic level), *df* = 2 (stimulation condition), *df* = 6 (animals), and *df* = 4 (interaction of anesthetic level-stimulation). The significance level is α = 0.05 and the statistically significant results are indicated with bold font.

**FIGURE 5:**
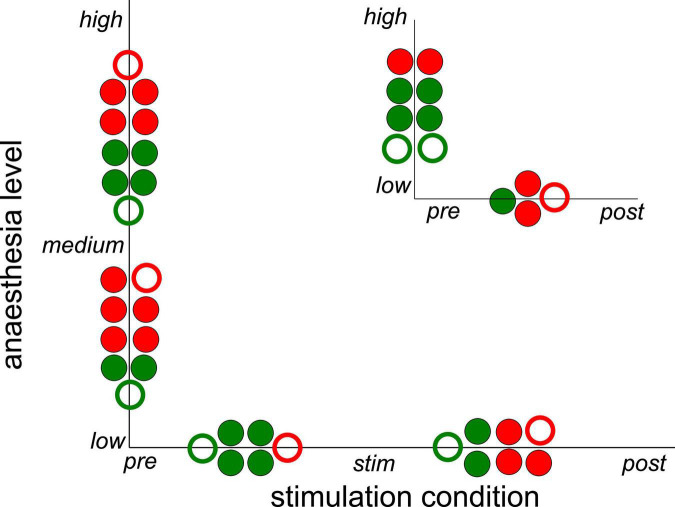
Significant impact on synchronization of stimulation condition and anesthetic level in single animals. Circles between two conditions or levels indicate statistically significant differences between median Phase Locking Values (PLVs) in channel clusters C2 and C3 in single animals. Full and empty circles indicate data computed for slow oscillations and γ-frequency band, respectively. The colors indicate increased (red) and decreased (green) synchronization. The significance level is α = 0.05. The plot summarizes results contained by Supplementary Tables 1, 2.

Subsequent statistical testing for the effects of stimulation condition and anesthetic level on synchronization (Supplementary Tables 1, 2) reveal further insights. Here, we have tested different pairs of stimulation conditions and different pairs of anesthetic levels. Figure 5 summarizes the statistically significant results which reveal that stimulation tends to decrease synchronization while post-stimulation activity tends to exhibit stronger synchronization than during stimulation. Comparing the pre-stimulation and post-stimulation phase, we find an essential synchronization enhancement. This may be explained by stimulus-induced synaptic plasticity that has been observed previously (Stauch et al., 2021). Moreover, increasing the administered anesthetic concentration from low to medium levels enhances the synchronization level, whereas further anesthetic concentration yields a weaker synchronization enhancement. The comparison of synchronization between high to low anesthetic concentration indicates a desynchronization, which is surprising.

**FIGURE 6:**
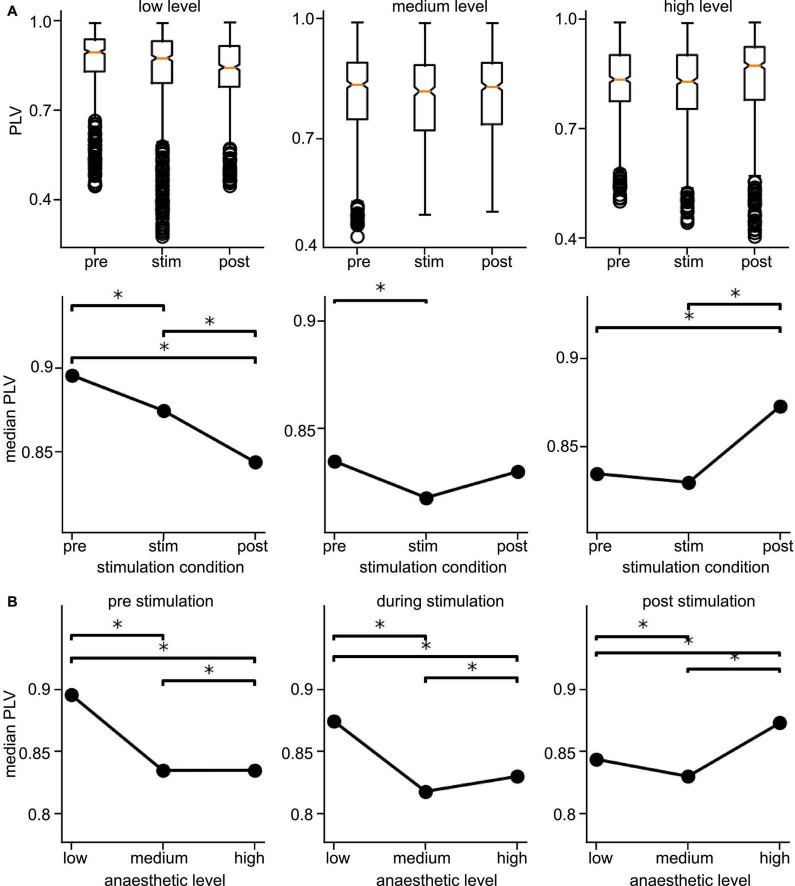
Distribution of Phase Locking Values (PLVs) and of their significantly different median values in slow oscillations. **(A)** Box plots of PLVs (top panels) and their median values (bottom panels). *Statistically different median values between stimulation conditions are indicated by horizontal starred brackets. **(B)** Statistically different medians between anesthetic levels are indicated by horizontal starred brackets. PLV values were taken from channel clusters C2 and C3, significance level α = 0.05 with Mann–Whitney U-test based on data from all animals.

**FIGURE 7:**
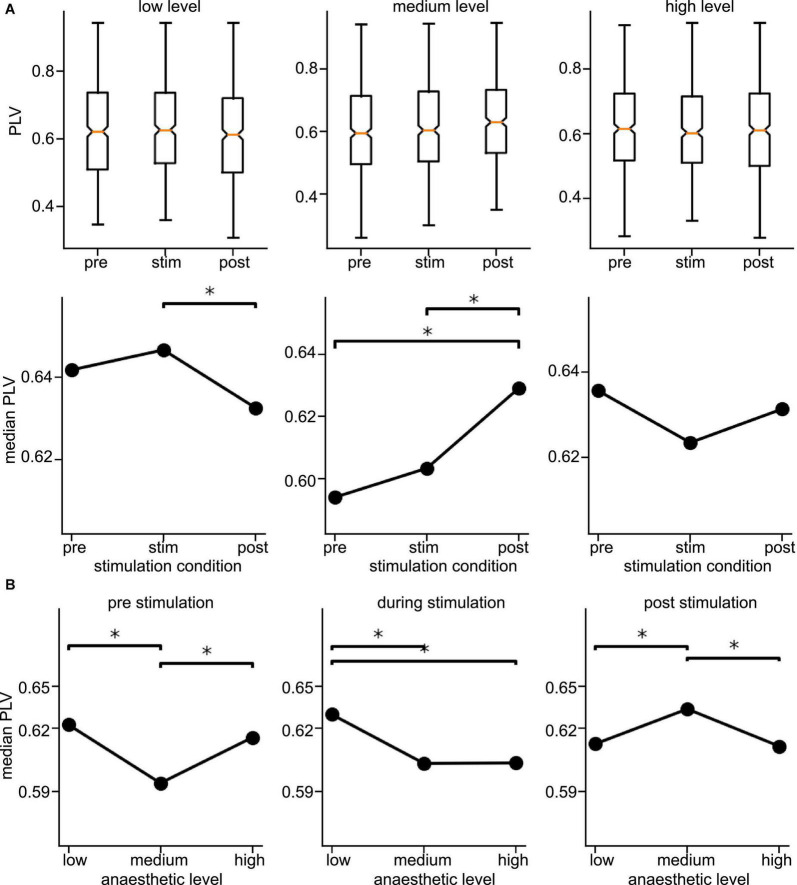
Distribution of Phase Locking Values (PLVs) and their significantly different median values in the γ-frequency band. **(A)** Box plots of PLVs (top panels) and their median values (bottom panels). *Statistically different median values between stimulation conditions are indicated by horizontal starred brackets. **(B)** Statistically different medians between anesthetic levels are indicated by horizontal starred brackets. PLV values were taken from channel clusters C2 and C3, significance level was α = 0.05 with Mann–Whitney U-test based on data pooled over all animals.

**FIGURE 8:**
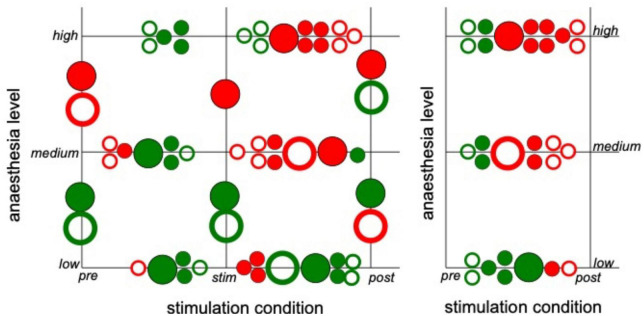
Strong interaction between anesthetic level and stimulation condition. The plot summarizes statistically significant synchronization differences between stimulation condition and anesthetic level. Each large circle denotes a statistically significant results from animal averages (taken from Figures 6, 7) and each small circle denotes a statistically significant single animal result (taken from Supplementary Figure 1). Full and empty circles represent results in slow oscillations and γ-frequency band, respectively, red and green colors indicate enhanced and diminished synchronization, respectively.

Since the latter results just shed some light on synchronization under anesthesia and stimulation, we attempted to reveal distinct effects by statistical tests over all animals (i) among stimulation conditions (before, during, and after) at each anesthetic level separately and (ii) among anesthetic levels (low, medium, and high) at each stimulation condition separately (Figure 6 for slow oscillations and Figure 7 for the γ-frequency band). We observe a strong heterogeneous interaction among stimulation condition and anesthetic level. At low anesthetic concentration, stimulation diminishes synchronization, and this effect is retained at post-stimulation. Increasing the anesthetic level inverts the picture, so that stimulation gains the tendency to enhance synchronization. We observe the strongest enhancement of synchronization at high anesthetic concentration in the post-stimulation phase. This is confirmed by statistical tests in individual animals (Supplementary Figure 1) and summarized in Figure 8.

### 3.2. Underlying structural network

**FIGURE 9:**
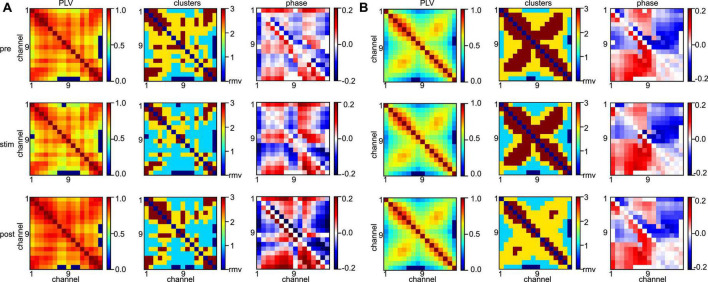
Different network synchronization in slow oscillations and γ-band activity. **(A)** Connectivity maps (left column), synchronization clusters C1 (cyan), C2 (yellow), and C3 (red) of channel pairs (center column), and phase differences between channels (right column) before stimulation (top row), during stimulation (center row), and after stimulation (bottom row) in slow oscillations. **(B)** Connectivity maps, synchronization clusters and phase differences in the three stimulation conditions in γ-band activity. Data has been taken from subject #7.

Statistical tests revealed that the synchronization level depends heavily on the subject and the frequency band under study. To learn more about the differences between animal data, Figure 9 shows the connectivity maps (left column) of subject #7 for all stimulation conditions (rows) and both frequency bands. We observe a rather disordered connectivity pattern in slow oscillations (A) as opposed to a pronounced cross-like structure in the γ-frequency band (B). The corresponding synchronization cluster distributions (center column) illustrate well this pattern difference. High degrees of synchronization (dark red) are surrounded by medium degrees of synchronization (yellow) in the γ-frequency band, while slow oscillations exhibit mixed and disordered synchronization pattern. This differences in connectivity pattern between the frequency bands correspond well to the connectivity structure of phase differences (right column). There one observes a clear increase in phase differences of certain channel pairs in the γ-frequency band (the *square* pattern in the figure). These pairs indicate connections between channels #1 and #8 and channels #9 and #16, i.e., channels capturing the activity in areas PtA and V2. Since increased phase differences reflect an increase in interaction delay between the channels, this pattern together with the results shown in the center column indicates a strong delay in synchronization between PtA and V2 in the γ-frequency band. However, we point out that the finite sampling rate of 500 Hz yields a phase value resolution of Δϕ≈ (1 Hz/500 Hz) ⋅ 2π≈ 0.01 for slow oscillations and Δϕ≈ (40 Hz/500 Hz) ⋅ 2π≈ 0.5, i.e., phase difference values of 0.1 as seen in the γ-frequency band are below the phase resolution and hence should be interpreted with care. Although statistical tests on the significance of phase differences were performed (not shown), they did not yield significant phase differences between stimulation conditions and anesthetic levels. Typical connectivity patterns in a different subject are shown in Supplementary Figure 2 and confirm the interpretations qualitatively.

**FIGURE 10:**
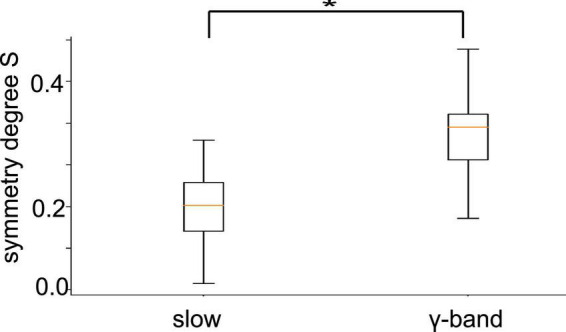
Symmetry between PtA and V2 connections. Symmetric synchronization (defined in section “2. Materials and methods”, Eq. 1) is significantly more prominent in the γ-frequency than in low-frequency oscillation (*p* < 10 – 8, α = 0.05). The horizontal bar with the star denotes statistically different median values between both frequency bands.

To quantify the visual impressions of synchronization differences in slow oscillation and in γ-band activities, we have considered the degree of synchronization symmetry between channels in PtA and V2 (cf. section “2. Materials and methods”). High values reflect strong symmetry between opposite synchronized channel pairs. Figure 10 shows box plots of symmetry degrees in both frequency bands. γ-Band activity is significantly more symmetrically synchronized (*p <* 10^–8^) than slow oscillations, i.e., the channels have a more regular spatial synchronization structure in the γ-frequency band than in slow oscillations. This holds over all animal subjects and experimental conditions.

**FIGURE 11:**
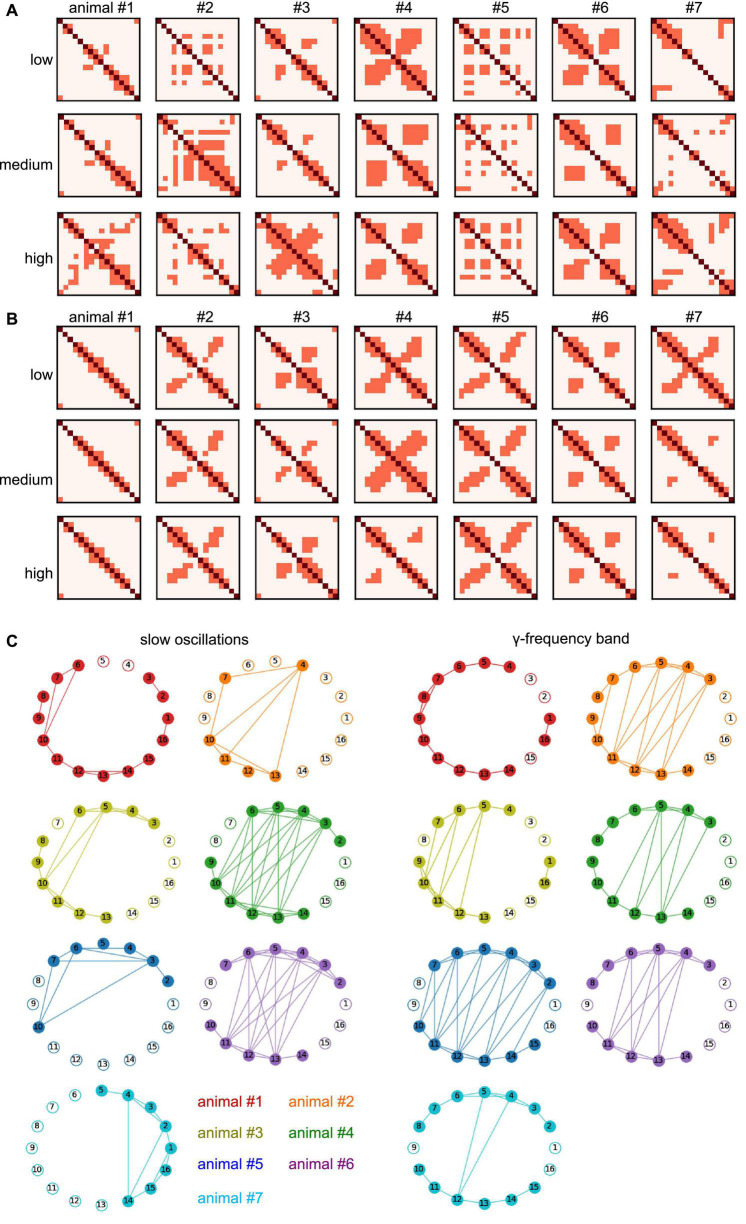
Invariant connectivity at all anesthetic levels and animal subjects. **(A)** Mutual connection maps subject to anesthetic level and animal subject in slow oscillations. Diagonal pixels (dark red) denote self-interactions, non-diagonal pixels (light red) denote maximum synchronization connections (cf. cluster 3 in Figure 9) which occur in all stimulation conditions. Rows provide results at different anesthetic levels, results from different animal subjects are given in columns. **(B)** Mutual connection maps in γ-band activity. **(C)** Invariant networks which share maximum synchronization in stimulation conditions and anesthetic level, i.e., mutual connections in panels **(A,B)** in all anesthetic levels. Different colors encode different animal subjects. Electrodes 1–8 and 9–16 reside in separate cortical regions. ^#^Denotes the animal number.

In addition to the experimental conditions and the frequency band, the degree of synchronization heavily depends on the animal subject (as already seen in Table 1). To better understand the difference in synchronization between animals, we have taken a closer look at the invariant connectivity, i.e., the mutual connectivity which is present at, and therefore independent of, different experimental conditions. Figures 11A, B display the connectivity, which is present in all stimulation conditions for different anesthetic levels and animal subjects. Apart from the diagonal line which represents trivial self-connections, a strong cross-like structure dominates the γ-frequency band (Figure 11B) reflecting symmetric nearest neighbor interactions (cf. Figures 2, 9, 10). This pattern in the γ-band is statistically different from the slow oscillation patterns as shown in Figure 10. Moreover, the strong synchronization, which is present in all stimulation conditions and all anesthetic levels, reflects connections that are independent of experimental conditions and thus called *invariant*. The corresponding networks that represent such invariant connections are shown in Figure 11C for different animals. The connectivity patterns are different between the two frequency bands and to some extent, among the animals. Despite this variation, both within-region and between-region connectivity can be recognized. Anatomical connections across the visual hierarchy, and by inference also V2-PtA, are known to be mirroring the retinotopic locations along the mediolateral region boundary. This is clearly reflected in the interregional functional connection pattern. Within-region connectivity is distinct and apparently strong in most animals, especially in the γ-band.

### 3.3. Information content

Synchronization may reflect FC and quantifies the degree time series and their underlying populations share information in a certain frequency band. However, it does not quantify how much and what type of information is shared or stored. Since previous studies indicated that the amount of stored, available, and transferred information change under anesthesia (Wollstadt et al., 2017), we were interested how stimulation and anesthesia affect information in the visuoparietal network involved in higher order processing of information. Table 3 provides changes of AIS between three combinations of stimulation and anesthesia level in all animal subjects. In invariant networks the actively stored information is lower after stimulation at low anesthesia level (*pre-low post-low*) and increases by increasing the anesthesia level after stimulation (*post-low post-high*). Effectively, this biphasic change of AIS (first increase and then decrease by stimulation in deeper anesthesia) balance each other and, as a result, the AIS in the pre-stimulation state at low anesthesia level (*pre-low*) and post-stimulation state at high anesthesia level (post-low) are not distinguishable from each other (*p* = 0.8). Hence, stimulation and anesthesia have a different impact in invariant networks. To our best knowledge, this has not been found before. Conversely, in varying networks, stimulation does not affect the AIS at low anesthesia level (*p* = 0.9) but increases it at deeper anesthesia. This result is in accordance with a previous anesthesia study (Wollstadt et al., 2017).

**TABLE 3:** Statistical inference of active information storage (AIS) for invariant and varying networks.

Invariant			Varying		
**Conditions**	**Mean diff.**	***p*-value**	**Conditions**	**Mean diff.**	***p*-value**
Pre-low post-high	-0.009	0.800	**Pre-low post-high**	**0.045**	**0**.**007**
**Post-low post-high**	**0**.**041**	**0.014**	**Post-low post-high**	**0.040**	**0**.**002**
**Pre-low post-low**	-**0**.**050**	**0.002**	Pre-low post-low	0.005	0.9

A first two-way ANOVA (not shown) over animals and combined condition pre-stimulation/low anesthesia level (pre-low), post-stimulation/low anesthesia level (post-low) and post-stimulation/high anesthesia level (post-high) shows significant differences between conditions and animals (*p* < 0.001) in both invariant and varying networks. Here, an invariant network considers all network nodes in a single animal subject in Figure 11, which are shared between both frequency bands. The table shows results of subsequent *post-hoc* pair-wise Tukey tests revealing a significant differences of AIS between the combined experimental conditions. The family-wise error rate is ∝ = 0.05,the statistically significant results are indicated with bold font. Supplementary Figures 3–6 provide corresponding AIS-values in the invariant network and the varying network. We mention, that different values of parameter k yielded different values of AIS (not shown), but the qualitative results were identical.

## 4. Discussion

In the current work we examined the interaction of PnO stimulation and desflurane anesthesia to better understand how they modulate spontaneous information processing in a local cortical network. Since the PnO plays a central role in the RAS and is one of the key targets of general anesthetics (Jones, 2003; Lydic and Baghdoyan, 2005), we raised the question how it contributes to cortical arousal modulation under anesthesia.

### 4.1. Interaction of brainstem stimulation and anesthesia

Brainstem stimulation affects the synchronization of LFP signals between area PtA and V2 as a function of the anesthetic level. At low anesthetic level, brainstem stimulation diminishes PtA-V2 synchronization. Increasing the anesthetic level inverts the picture so that brainstem stimulation enhances synchronization (Figure 8). Moreover, the maximum synchronization enhancement emerges at high anesthetic level and in the post-stimulation period.

These findings may be interpreted in the context of neuronal activity. At low anesthetic concentrations neurons fire asynchronously and thus desynchronize the corresponding LFPs. This state is characterized by a relatively large number of distinct brain states (Pillay et al., 2014b). At higher anesthetic concentrations, inhibition is enhanced (Alkire et al., 2008) and some neurons may cease to fire, which leads to stronger synchronization in the LFPs. This opposite impact of brainstem stimulation and anesthesia resembles a recent hypothesis on the role of brainstem activity as part of the reticular arousal system (RAS) in the context of general anesthesia under propofol (Hutt et al., 2018). The authors of the work had proposed supported by extended model simulations that enhanced RAS activity had an impact similar to that of reducing the anesthetic concentration, i.e., stimulation and anesthetics yielded the opposite effects. The authors hypothesized that RAS activation enhances endogenous fluctuations and renders these fluctuations more irregular. This hypothesis could also explain experimentally observed features of frontal and occipital EEG spectral power under anesthesia. In the light of these results, the opposite stimulation-anesthesia effect was also found in the basal forebrain-paralimbic network under brainstem stimulation and isoflurane anesthesia (Pillay et al., 2014a). The authors of this work found that the brainstem stimulation increased the observed activities’ irregularity opposite to the anesthesia impact. Our experimental results are in line with these findings supporting the hypothesis of the brainstem’s function to tune the regularity of endogenous fluctuations. The opposing effects of stimulation and anesthesia probably apply to other stimulation locations and anesthetics including desflurane. For instance, desflurane is known to reduce motor-evoked activity under desflurane and propofol anesthesia (Malcharek et al., 2015). Moreover, thalamic electric stimulation has been shown to reverse propofol anesthesia in monkeys (Bastos et al., 2021).

In addition to the opposite effects of stimulation and anesthesia, AAS stimulation had a long-term impact on post-stimulation (Figure 5), as expressed by the stimulus-induced desynchronization and synchronization at low and high anesthetic concentrations, respectively. This is in line with stimulus-induced plasticity found previously in several cortical areas (Di Pino et al., 2014), such as the visual cortex (Stauch et al., 2021) and the motor cortex (Sowman et al., 2014). The finding is also consistent with anesthesia-induced neural plasticity (Hogan et al., 2020) in accordance with the impairment of memory and long-term potentiation in rats under desflurane aesthesia (Tojo et al., 2019).

### 4.2. Slow oscillations are more sensitive to stimulation and anesthesia than *γ-*activity

The effects of stimulation and anesthesia on the spectral power of both slow oscillations and γ-activity were notably dependent on the animal subject (Figure 3). This is in line with previous studies showing a relatively high variability of LFP power among animals (Lee et al., 2020). While the effects of stimulation and anesthesia on spectral power were rather heterogeneous in both frequency bands (Figure 3), their effect on synchronization were distinguishable in slow oscillations and γ-activity. We have seen that synchronization of slow oscillations varies with the experimental stimulation condition and the anesthesia level (Table 2), whereas experimental conditions hardly affect γ-activity synchronization, cf. Table 3, see also Figure 8. This insensitivity in γ-activity to stimulation and anesthesia may be reflected in the strong phase synchronization symmetry seen in Figure 10. This symmetry reflects the spatial regularity of connections in the visuoparietal network, i.e., how similar visual and parietal connections are. This regularity is stable over stimulation and anesthesia conditions in the γ-band only, whereas it is not present in slow oscillations. Consequently, the regularity seems to originate from a unique γ-band mechanism. A possible explanation may be a coherent propagation of γ-activity over the visuoparietal network observed experimentally before in the posterior cortical areas (Bahramisharif et al., 2013) and in the visual cortex (Gabriel and Eckhorn, 2003). Prior studies found that various inhalational anesthetics including desflurane did not attenuate, sometimes even increased, γ-power in rat sensorimotor, parietal, and visual cortex (Vanderwolf, 2000; Imas et al., 2005a). However, the same anesthetics suppressed frontal-parietal, parietal-occipital and frontal-occipital directed connectivity in the γ-band during visual stimulation (Imas et al., 2005b). Thus, several opposing factors may contribute to the inconsistent change in γ-activity across different anesthetic and arousal conditions.

### 4.3. Differential impact on information storage by stimulation and anesthesia

Stimulation appears to diminish AIS, i.e., the information stored in the network. An opposite raise of stored information is observed for larger anesthesia levels, cf. Table 3. This holds true in so-called *invariant networks*. Such networks are animal-specific and include strong connections which are present in all experimental conditions and both frequency bands under study. We argue that they may be invariant in the sense that they are independent of any specific functions induced by stimulation and anesthesia. Interestingly, stored information in complement networks, i.e., *varying networks*, is not affected by stimulation. Larger anesthesia levels raise the stored information in accordance to previous experimental findings in ferret visual and pre-frontal cortex under isoflurane anesthesia (Wollstadt et al., 2017).

### 4.4. Limits and perspectives

The main finding, that brainstem stimulation and anesthesia level interact and induce activity resembling neural plasticity in a state-dependent way, has not been investigated further in detail. For instance, investigating the temporal evolution of plasticity could provide additional insights into the stimulus-anesthesia interaction. Since such a study would exceed the limits of the present article, we refer the reader to future work.

Subject group analysis of the data was somewhat limited by the variability in stimulation induced LFP spectral changes and in FC patterns. The variable and sometimes unexpected effect of anesthesia on cortical *γ-*activity has been noted before (Imas et al., 2005a) that may generalize to stimulation conditions. More extensive interregional FC (electrodes 1–8 vs. 9–16) was observed in some animals than in others, although the within-region connectivity was more consistent. It is possible that inadvertent variations in electrode positioning occurred despite the electrodes’ stereotaxic placement. Histological verification of the final electrode locations may help sort out possible errors in the future. However, normal animal to animal anatomical variation could also account for some of the discrepancies in FC.

Our study used the inhalational anesthetic desflurane that has the characteristic of rapid equilibration in tissues, which aids the conduct of experiments. Other types of anesthetics with different pharmacology may confer different changes in cortical LFP spectra and FC (Lee et al., 2013). Nevertheless, desflurane represents the neurophysiological action of the general class of inhalational anesthetics commonly acting on GABA_*A*_ receptors (Alkire et al., 2008). The effect of intravenous anesthetics may be investigated in the future.

It will be also interesting to investigate how stimulation and anesthesia impact information processing and storage in PtA and V2. Previously, Pillay et al. (2014a) found that PnO stimulation in rats augmented the information complexity of neuronal interactions in PtA more than in V2 suggesting that higher order association cortex is under a stronger modulation by anesthetics and the arousal system than modality-specific sensory cortex. However, the opposite outcome was observed during visual stimulation suggesting a preferential enhancement of visual information processing in V2 during arousal by brainstem stimulation. Since LFPs more faithfully reflect the collective behavior of neuron populations than single units, it will be of interest in the future to examine the dependence of LFP-derived information capacity on anesthetic/arousal levels during sensory stimulation.

## Data availability statement

The original contributions presented in this study are included in the article/Supplementary material, further inquiries can be directed to the corresponding author.

## Ethics statement

This animal study was reviewed and approved by the Institutional Animal Care and Use Committee of the Medical College of Wisconsin (Milwaukee, WI, United States).

## Author contributions

AH and AGH conceived the work and wrote the manuscript to equal parts. AH performed the data analysis. Both authors contributed to the article and approved the submitted version.
