# Supplementary material for: Arousal system stimulation and anesthetic state alter visuoparietal connectivity
Source: Front Syst Neurosci. 2023 Apr 17;17:1157488. doi: 10.3389/fnsys.2023.1157488 (PMC10150228; doi:10.3389/fnsys.2023.1157488)
Supplement: Supplementary file 1 [file Data_Sheet_1.PDF]

Supplementary Material

Arousal system stimulation and anesthetic state alter visuo-parietal connectivity

A Hutt *, AG Hudetz

*** Correspondence: Axel Hutt**: axel.hutt@inria.fr

1 Supplementary Data

The Table S1 and S2 denote the ANOVA results on PLV values in single animals. The factors are either the stimulation conditions or the anaesthetic levels. We observe that slow oscillations exhibit a larger number of significant differences between experimental conditions than in the γ−frequency band. Moreover, results are quite different in different animals.

animal	conditions	mean diff	p-value	conditions	mean diff	p-value
#1	post-pre	−0.0143	0.4266	**high-low**	**−0.0759**	**0.001**
	**post-stim**	**0.0369**	**0.0035**	**high-medium**	**−0.0591**	**0.001**
	**pre-stim**	**0.0511**	**0.001**	**low-medium**	**−0.135**	**0.001**
#2	post-pre	−0.0025	0.9	**high-low**	**−0.0576**	**0.001**
	post-stim	0.0025	0.7159	**high-medium**	**−0.1105**	**0.001**
	pre-stim	0.0077	0.5112	**low-medium**	**−0.0529**	**0.001**
#3	**post-pre**	**−0.0539**	**0.001**	**high-low**	**−0.0952**	**0.001**
	**post-stim**	**−0.0370**	**0.001**	**high-medium**	**−0.0533**	**0.001**
	pre-stim	0.0169	0.0615	**low-medium**	**0.0419**	**0.001**
#4	post-pre	−0.0209	0.1088	**high-low**	**0.0361**	**0.001**
	**post-stim**	**−0.0323**	**0.0045**	**high-medium**	**0.1596**	**0.001**
	pre-stim	0.0113	0.5209	**low-medium**	**0.1235**	**0.001**
#5	post-pre	−0.0115	0.3188	**high-low**	**−0.0488**	**0.001**
	post-stim	0.0182	0.0561	**high-medium**	**0.096**	**0.001**
	**pre-stim**	**0.0297**	**0.001**	**low-medium**	**0.1448**	**0.001**
#6	**post-pre**	**0.0251**	**0.001**	high-low	0.0092	0.3264
	**post-stim**	**0.0473**	**0.001**	**high-medium**	**0.0343**	**0.001**
	**pre-stim**	**0.0222**	**0.0015**	**low-medium**	**0.0251**	**0.001**
#7	**post-pre**	**0.0196**	**0.001**	high-low	0.0184	0.0011
	**post-stim**	**0.0367**	**0.001**	**high-medium**	**0.052**	**0.001**
	**pre-stim**	**0.017**	**0.0053**	**low-medium**	**0.0336**	**0.001**

Table S1 Inference test in single animals for slow oscillations. *Two-way ANOVA has been applied, bold font denotes statistically significant results with significance level α = 0.05.*

animal	conditions	mean diff	p-value	conditions	mean diff	p-value
#1	post-pre	0.0081	0.679	high-low	0.0052	0.8586
	**post-stim**	**−0.0294**	**0.0177**	**high-medium**	**−0.0262**	**0.0399**
	**pre-stim**	**−0.0375**	**0.0013**	**low-medium**	**−0.0314**	**0.0063**
#2	post-pre	−0.0246	0.1068	high-low	0.0045	0.9
	**post-stim**	**0.0311**	**0.0207**	high-medium	−0.0082	0.7562
	**pre-stim**	**0.0557**	**0.0010**	low-medium	−0.0127	0.5334
#3	post-pre	0.0064	0.8994	**high-low**	**−0.0565**	**0.001**
	post-stim	−0.0224	0.3155	high-medium	−0.0092	0.7872
	pre-stim	−0.0289	0.1294	**low-medium**	**0.0473**	**0.005**
#4	post-pre	−0.0208	0.2855	**high-low**	**−0.0349**	**0.0322**
	post-stim	−0.0197	0.3382	high-medium	−0.0241	0.1914
	pre-stim	0.0011	0.9	low-medium	0.0109	0.694
#5	post-pre	−0.0038	0.9	high-low	−0.0126	0.4379
	post-stim	−0.0019	0.9	high-medium	−0.0005	0.9
	pre-stim	0.0019	0.9	low-medium	0.0122	0.4632
#6	post-pre	0.0097	0.7026	high-low	−0.0035	0.9
	post-stim	0.0092	0.7269	high-medium	−0.0002	0.9
	pre-stim	−0.0005	0.9	low-medium	0.0033	0.9
#7	**post-pre**	**0.0349**	**0.001**	high-low	0.0195	0.0692
	post-stim	0.0247	0.149	**high-medium**	**0.0354**	**0.001**
	pre-stim	−0.0102	0.4612	low-medium	0.0159	0.1590

Table S2 Inference test in single animals for the γ-frequency band. *Two-way ANOVA has been applied, bold font denotes statistically significant results with significance level α = 0.05.*

Figure S1 shows the medians in all experimental conditions, all animals and both frequency bands. We observe a rather heterogeneous animal-dependent impact of experimental conditions on the synchronization.

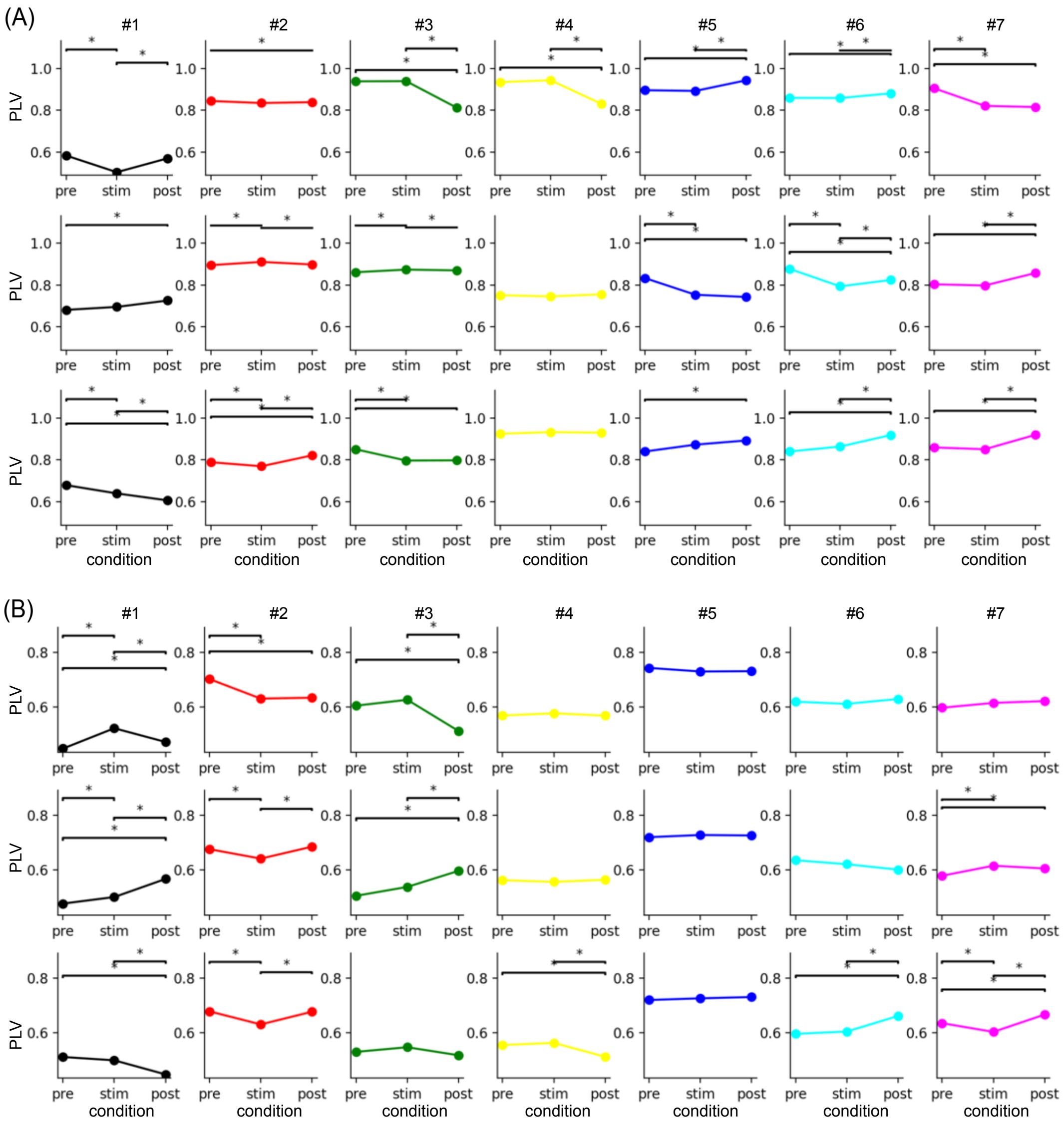

Figure S1 Median PLVs in single animals, in all stimulation conditions and all anaesthetic levels. *(A) Results in slow oscillations and (B) in the γ–frequency band. Columns provide results in animals #1-#7, rows at low (top), medium (center) and high (bottom) anesthetic level. Colors distinguish results of different animals in correspondence to the figure color encoding in the main manuscript.*

The connectivity maps in Figure S2 illustrate synchronized spatial networks in two animals in all experimental conditions.

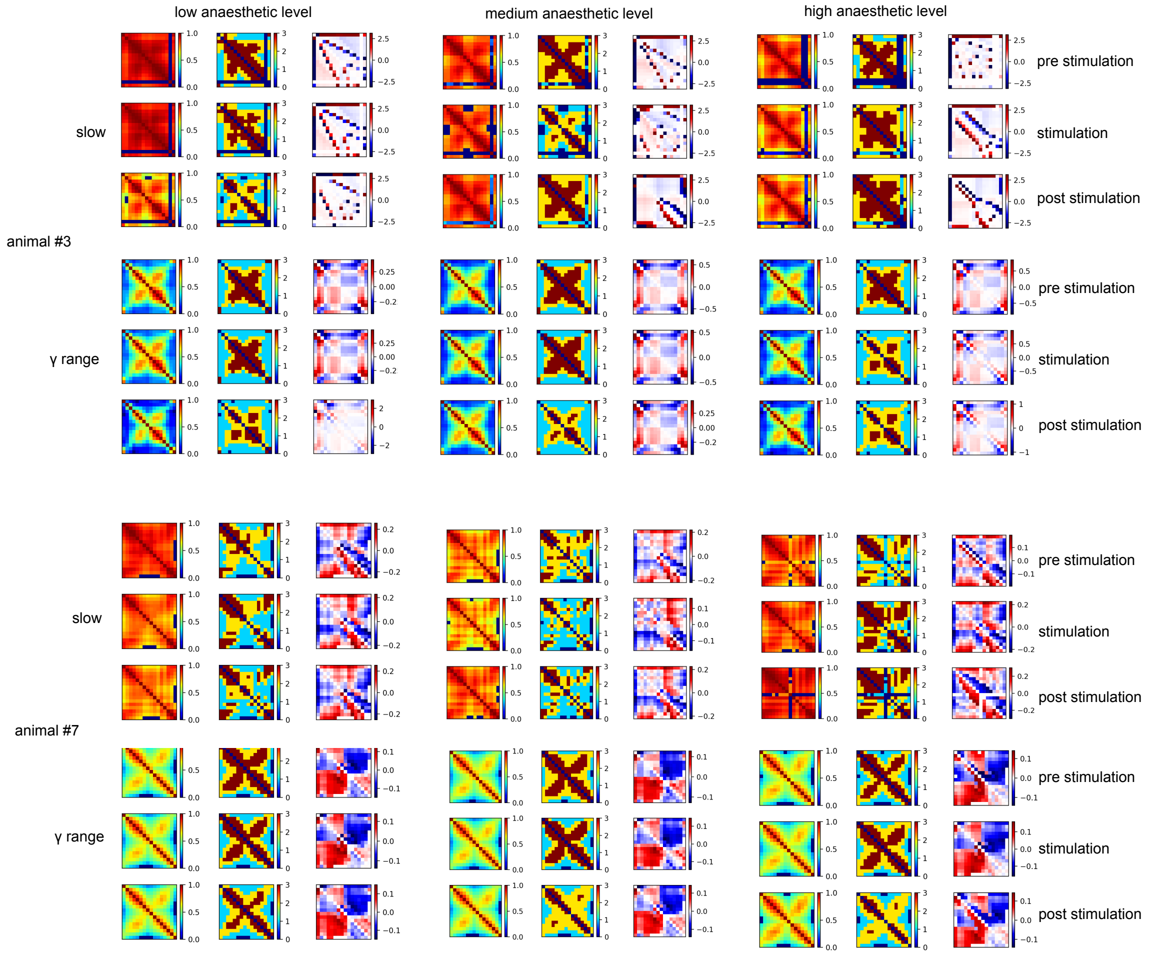

Figure S2 Connectivity maps, synchronization clusters and phase difference maps for all experimental conditions and both frequency bands in animal subject #3 and #7. *Synchronization is stronger in slow oscillations (red color) compared to the γ−band synchronization (green and yellow colors). We observe a cross-like structure in the γ−frequency band indicating a symmetry in connections in the parietal and visual cortex electrode channels. This symmetry is much less pronounced in slow oscillations.*

Finally, Figures S3-S7 provide the AIS in each channel in non-functional and functional networks in all animals.

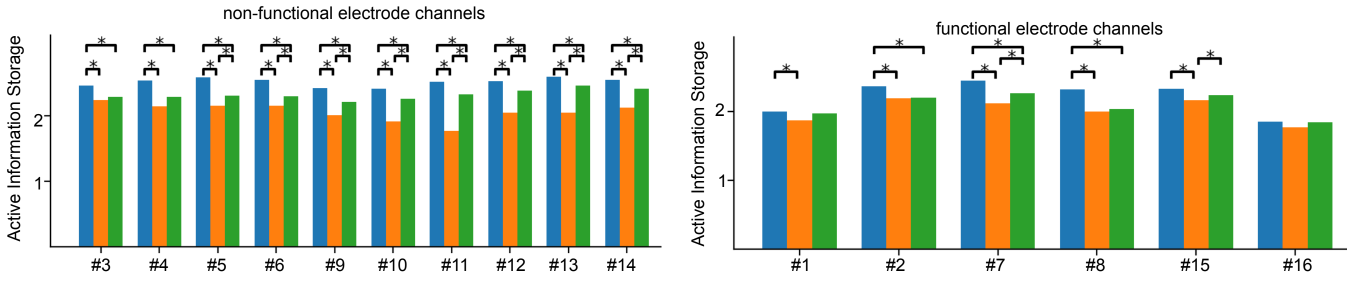

Figure S3 Active Information Storage (AIS) in non-functional and functional channel in animal #4. *Significant differences (non-parametric pair-wise Tukey test, significance threshold was α=0.05) are denoted by bars and stars. The experimental conditions under study were pre-stimulation/low anesthesia level (blue), post-stimulation/low anesthesia level (orange) and post-stimulation/high anesthesia level (green).*

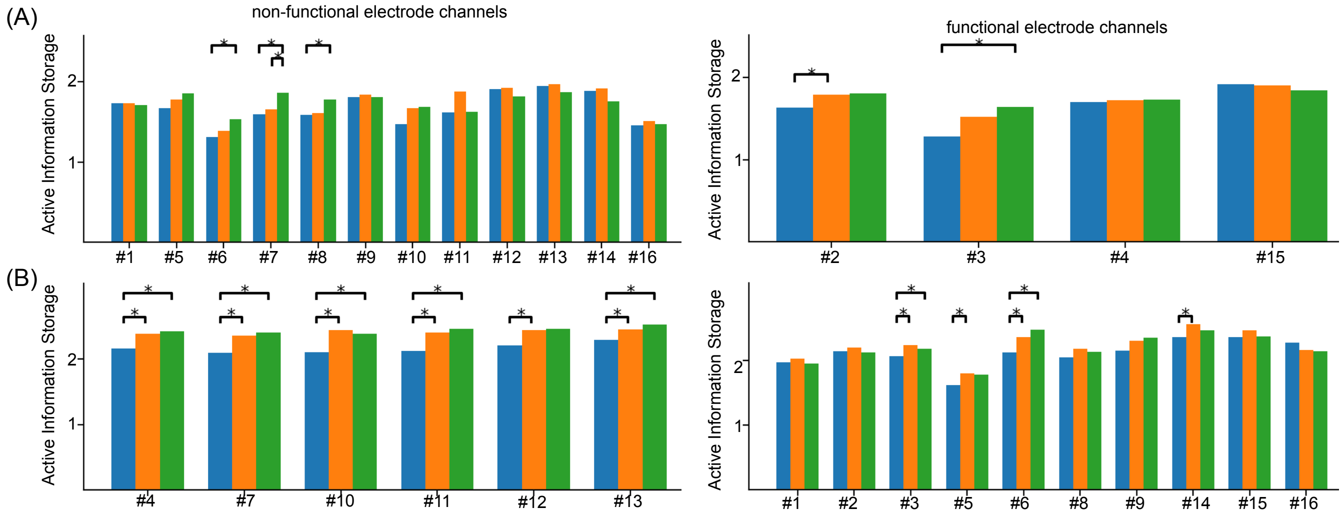

Figure S4 Active Information Storage (AIS) in non-functional and functional channel in animal #1 (A) and #2 (B)*. Significant differences (non-parametric pair-wise Tukey test, significance threshold was α=0.05) are denoted by bars and stars. The experimental conditions under study were pre-stimulation/low anesthesia level (blue), post-stimulation/low anesthesia level (orange) and post-stimulation/high anesthesia level (green).*

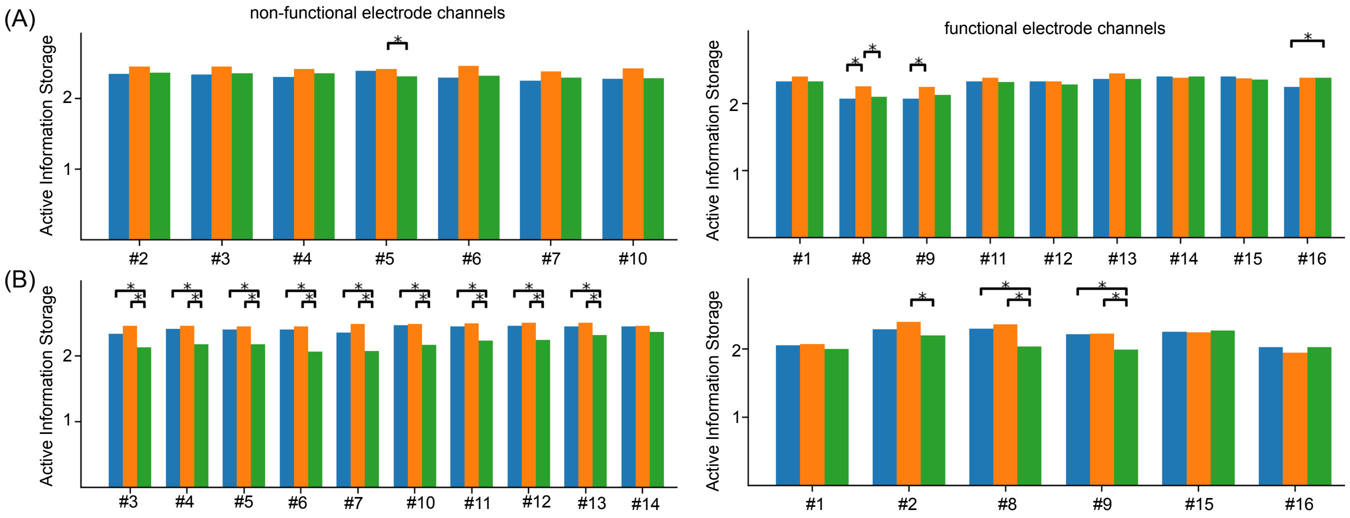

Figure S5 Active Information Storage (AIS) in non-functional and functional channel in animal #5 (A) and #6 (B). *Significant differences (non-parametric pair-wise Tukey test, significance threshold was α=0.05) are denoted by bars and stars. The experimental conditions under study were pre-stimulation/low anesthesia level (blue), post-stimulation/low anesthesia level (orange) and post-stimulation/high anesthesia level (green).*

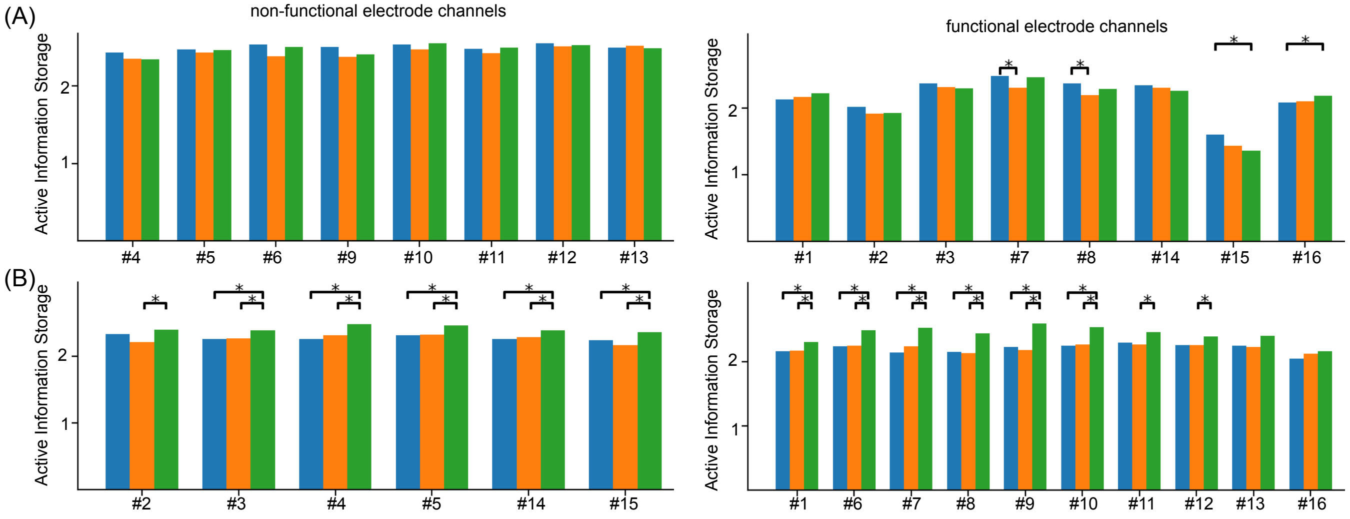

Figure S6 Active Information Storage (AIS) in non-functional and functional channel in animal #3 (A) and #7 (B*). Significant differences (non-parametric pair-wise Tukey test, significance threshold was α=0.05) are denoted by bars and stars. The experimental conditions under study were pre-stimulation/low anesthesia level (blue), post-stimulation/low anesthesia level (orange) and post-stimulation/high anesthesia level (green).*
